# Vascular Guidance: Microstructural Scaffold Patterning for Inductive Neovascularization

**DOI:** 10.4061/2011/547247

**Published:** 2010-12-01

**Authors:** Daniel Muller, Harvey Chim, Augustinus Bader, Matthew Whiteman, Jan-Thorsten Schantz

**Affiliations:** ^1^Department of Plastic, Reconstructive and Handsurgery, Klinikum rechts der Isar, Technische Universität München, 80333 München, Germany; ^2^Department of Plastic Surgery, Case Western Reserve University, Cleveland, OH 44106, USA; ^3^Division of Plastic Surgery and Bioengineering, National University of Singapore, Singapore 119077; ^4^Zentrum für Stammzellbiologie und Biotechnologie, Universität Leipzig, Germany; ^5^Peninsula Medical School, University of Exeter, Exeter EX4 4QJ, UK

## Abstract

Current tissue engineering techniques are limited by inadequate vascularisation and perfusion of cell-scaffold constructs. Microstructural patterning through biomimetic vascular channels within a polymer scaffold might induce neovascularization, allowing fabrication of large engineered constructs. The network of vascular channels within a frontal-parietal defect in a patient, originating from the anterior branch of the middle meningeal artery, was modeled using computer-aided design (CAD) techniques and subsequently incorporated into polycaprolactone (PCL) scaffolds fabricated using fused deposition modeling (FDM). Bone marrow-derived mesenchymal stem cells (MSCs) were seeded onto the scaffolds and implanted into a rat model, with an arteriovenous bundle inserted at the proximal extent of the vascular network. After 3 weeks, scaffolds were elevated as a prefabricated composite tissue-polymer flap and transferred using microsurgical technique. Histological examination of explanted scaffolds revealed vascular ingrowth along patterned channels, with abundant capillary and connective tissue formation throughout experimental scaffolds, while control scaffolds showed only granulation tissue. All prefabricated constructs transferred as free flaps survived and were viable. We term this concept “*vascular guidance,*” whereby neovascularization is guided through customized channels in a scaffold. Our technique might potentially allow fabrication of much larger tissue-engineered constructs than current technologies allow, as well as allowing tailored construct fabrication with a patient-specific vessel network based on CT scan data and CAD technology.

## 1. Introduction

A major limitation in clinical translation of tissue engineering techniques remains inadequate vascularisation, and hence perfusion of cell-scaffold constructs. In many studies using polymeric scaffolds in bone tissue engineering, tissue formation has been confined to the area immediately underneath the surface of the construct due to poor media perfusion of cells in the depths of the scaffold, resulting in impaired delivery of nutrients and growth factors, as well as hypoxia due to poor oxygenation. 

An early study with poly-L-glycolic acid (PLGA) foams found that the penetration depth of osseous tissue was restricted to 190–220 *μ*m from the surface of a tissue-engineered construct [1]. Subsequent strategies utilising bioreactors [2] and material modifications incorporating hydroxyapatite into polymer scaffolds [3] resulted in penetration depths of 3.7 mm and 1.4 mm, respectively. With the use of novel biomimetic scaffold architectures such as one mimicking trabecular bone [4], tissue reaching a depth of 10 mm was engineered. Most recently, our group and others reported the use of fused deposition modeling (FDM), a rapid prototyping technique, to engineer geometrically symmetric polycaprolactone (PCL) scaffolds with complete interconnectivity, allowing for osseous tissue formation throughout the entire cell-scaffold construct [5–7].

While these techniques might allow tissue formation throughout the entire extent of a small polymeric scaffold, they would not be sufficient to ensure adequate perfusion of a large tissue-engineered construct, which would be required for the vast majority of clinical applications. Examples of these include a calvarial bone flap for cranioplasty, or an engineered long bone segment to replace segmental extremity defects. 

We postulated that microstructural patterning through creation of biomimetic vascular channels following an open channel design within the polymer scaffold might allow natural inductive neovascularization, thereby creating a scaffold allowing excellent perfusion in its entirety. As a result, large complex engineered cell-scaffold constructs could potentially be created, representing a major step forward towards clinical application of bone tissue engineering techniques. For our clinical model, we chose the calvarial bone flap model.

## 2. Materials and Methods

### 2.1. Conceptualization and Design of Vascular Channels

**Figure 1:**
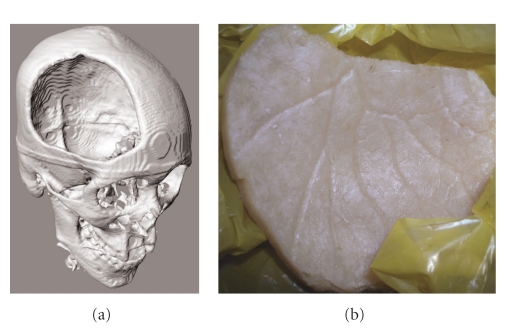
(a) Three-dimensional reconstruction of skull defect based on computed tomography (CT) scans of a patient who had a right frontoparietal craniectomy. (b) The arborizing course of the anterior branch of the middle meningeal artery can be observed on the internal aspect of the bone flap, providing a model for design of vascular channels.

The frontal-parietal bone flap elevated in a standard craniectomy results in a significant skull defect (Figure 1(a)). On the internal aspect of the bone flap, grooves caused by the middle meningeal artery can be seen (Figure 1(b)), which arborize in a branching pattern to perfuse the calvarium, together with the anterior and posterior meningeal arteries. Due to limitations of the computer-aided design (CAD) software used, only 3 factors could be edited for design of the vascularization pattern, which included the angle of branching, channel diameter, and space between tubes. 

**Figure 2:**
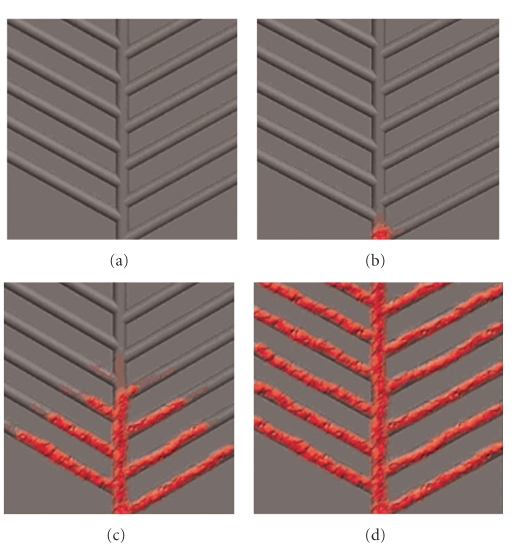
CAD model of design for vascular channels, with predicted pattern of blood flow. The model predicts orderly blood flow proceeding proximally from the source vessel to distal channels in a symmetric fashion.

Through analysis of the arborization pattern of the middle meningeal artery, we arrived at several rules and assumptions for design of vascular channels in our scaffold. These include the following: (1) angle of branching of channels should be around 45 degrees; (2) channel diameter was set at 1 mm due to mechanical constraints and ease of fabrication; (3) spacing between tubes was set at 1 mm. Spacing between capillaries in the human body has been reported to be 200 *μ*m, with a capillary diameter of around 50 *μ*m. Fabrication of scaffolds with a physiological channel spacing of 200 *μ*m would result in compromised mechanical strength, hence a tube spacing of 1 mm was used instead. The Pro/ENGINEER software (Parametric technology corporation, Needham, MA) was used to create a CAD model of the scaffold, together inclusive of vascular channels.  Figure 2 shows the resultant CAD model used for fabrication of vascular channels with the predicted blood flow pattern.

### 2.2. Fabrication of Polymer Scaffolds

**Figure 3:**
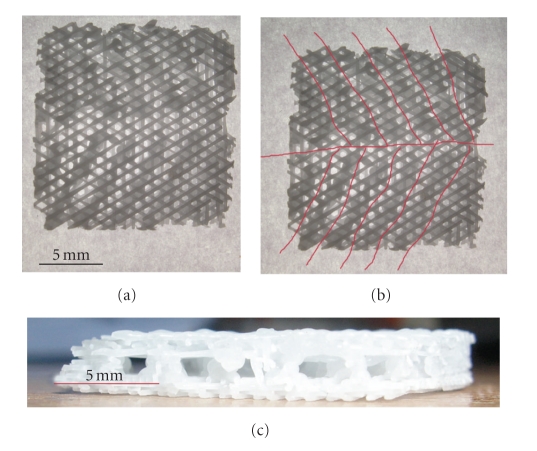
Fabricated PCL scaffold with incorporated vascular channels in arborizing pattern following an open channel design, based on course of middle meningeal artery in the calvarium. (a) Top view. (b) Red markings show branching vascular channels. (c) Side view.

Poly-e-caprolactone (PCL) of molecular weight (Mn) 80,000 (Sigma-Aldrich) was used, together with hydroxyapatite (HA), in a 9 : 1 ratio. The PCL-HA scaffolds were fabricated using fused deposition modeling (FDM), a rapid prototyping process, as previously described [8]. For this study, scaffolds measuring 18 × 18 × 3 mm were fabricated with a filament laydown pattern of 0, 60, and 120 degrees, and a porosity of 70%.  Figure 3(a) shows the top view of the fabricated scaffold, with red markings showing the branching vascular channels (Figure 3(b)).  Figure 3(c) shows the side view of the scaffold, with channel diameter of 1 mm. 

### 2.3. Cell Isolation and Culture—Preparation of Scaffolds

Porcine bone-marrow-derived mesenchymal stem cells (MSCs) were harvested using standard protocols [9]. Cells used were at passage two and maintained in complete DMEM + GlutaMAX I (Gibco, USA) [10% FBS + 1% Pen/Strep]. Cultures were passaged once a week and media changed every three days. 

Prior to cell seeding, scaffolds were washed with phosphate-buffered saline (PBS) and sterilized with 70% ethanol. Subsequently, scaffolds were seeded with an MSC-fibrin glue suspension. MSCs at a density of 1 × 10^6^ cells were initially suspended in fibrinogen (Tisseel, Baxter Biosciences, Germany) and then seeded homogenously onto PCL scaffolds. Thrombin was added to polymerize the mixture and create a fibrin glue simulating the extracellular matrix around cells.

### 2.4. Animal Surgery and Histological Analysis

**Figure 4:**
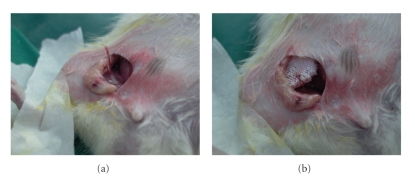
Rat groin model for investigation of vascular guidance. (a) Inferior epigastric vessels are isolated and ligated. (b) Inferior epigastric vascular pedicle is inserted into the proximal egress into the vascular channel network and secured with suture.

A 2 cm incision was made in the right groin of adult male immunodeficient CBH-ruul/+ nude rats, following which the inferior epigastric artery and vein were dissected out and ligated en masse (Figure 4(a)). The vascular pedicle thus created was then inserted into the proximal opening of the vascular channel network in the experimental scaffold (which had been previously seeded with MSCs) and secured with 4-0 Prolene (Ethicon, Sommerville, NJ) (Figure 4(b)). The scaffolds were then placed in a subcutaneous groin pocket, and the wound closed in layers. Control scaffolds were identical to experimental scaffolds in size, material, and laydown pattern and had MSCs seeded at the same density. However, these were placed in the contralateral groin without ligation or implantation of the inferior epigastric pedicle. Animals (*n* = 5) were sacrificed after 3 weeks, and specimens explanted for histological analysis with hematoxylin and eosin (H&E) staining. 

**Figure 5:**
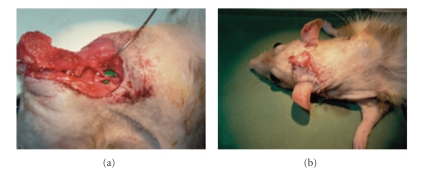
Explanted scaffolds after 3 weeks in vivo in rat groin model were transferred as composite tissue-polymer-free flaps to the neck. (a) End-to-side anastomosis of inferior epigastric artery to the common carotid artery (white marker). Inferior epigastric vein was also anastomosed in end-to-side fashion to the internal jugular vein. (b) After surgery with flap inset.

To investigate the hypothesis that the prefabricated scaffold constructs could be transferred via microsurgical technique as a free flap after 3 weeks *in vivo*, some of the explanted specimens (*n* = 3) were raised en bloc as part of a composite osteocutaneous-scaffold groin flap based on the inferior epigastric vessels. The inferior epigastric artery was then anastomosed using 10-0 Prolene (Ethicon, Sommerville, NJ) sutures via end-to-side technique to the common carotid artery (Figure 5(a)), while the inferior epigastric vein was anastomosed end-to-side to the internal jugular vein. The flap was inset into the scalp of the rat (Figure 5(b)).

Housing and feeding of the animals were done in accordance with standard animal care protocols. The study was approved by the Animal Welfare Committee, National University of Singapore and licensed by the National Institutes of Health's (NIH) *Guide for the Care and Use of Laboratory Animals.*

## 3. Results

**Figure 6:**
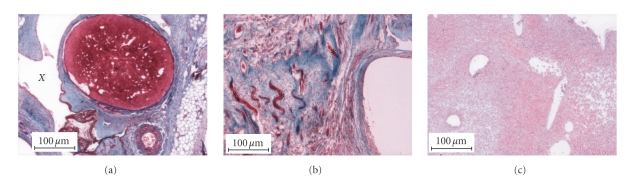
(a) Experimental scaffolds explanted after 3 weeks in vivo show evidence of blood vessel formation (red, marked ∗) with surrounding connective tissue (blue areas). These areas together represent a vascular channel that was prefabricated within the PCL-HA scaffold. Clear areas occupied by surrounding PCL-HA scaffold fibers are marked with an “X”. (b) Multiple capillaries (red, marked with ∗) are also seen in areas of connective tissue, indicating extensive neovascularization. (c) In contrast, control scaffolds only show granulation tissue without evidence of significant neovascularization.

H&E staining of experimental scaffolds 3 weeks after implantation showed formation of blood vessels in the vascular channels following the microstructural arborization pattern of the scaffold (Figure 6(a)), surrounded by connective tissue. In addition, there was evidence of angiogenesis in the connective tissue, with formation of multiple capillaries (Figure 6(b)). Angiogenesis was seen throughout the construct, permeating throughout the connective tissue. In contrast, for the control scaffolds, only granulation tissue was observed without evidence of neovascularization (Figure 6(c)).

Following transfer of prefabricated composite tissue-polymer groin flaps to the neck, the flaps were observed to be viable as observed clinically by good perfusion and capillary refill, as well as dopplerable arterial and venous signals.

## 4. Discussion

A piece of tissue with a volume exceeding a few cubic millimeters cannot survive by diffusion of nutrients but requires growth of capillaries for the supply of essential nutrients and oxygen [10]. Correspondingly, when a cell is more than 150–200 *μ*m from a blood vessel, it suffers from hypoxia and limitation of other nutrients because this is the maximum distance it can stay alive by diffusion only [11]. For tissue engineering to progress to widespread clinical application, therefore, creation of a microvascular network of capillaries and supporting macrovascular circulation is essential to support perfusion of large complex tissues and whole organs.

Early studies on flap prefabrication were focused on inducing formation of an intrinsic vascular supply in a block of tissue through staged microvascular transfer, in order to improve survival [12, 13]. However, reports of venous insufficiency following second-stage transfer as well as the technical complexity of these procedures have limited widespread use of prefabricated free flaps [14]. Subsequent efforts have been focused more on achieving vascularisation in *in vitro* models. These have included the use of an arteriovenous bundle, as was used in this study; or an arteriovenous loop to induce the formation of a network of vessels for prefabrication of skin flaps [15, 16].

In many of these studies, a vascularized chamber was used to study induction of tissue formation. In a different experimental context of an in vivo plastic chamber housing a poly (DL-lactic-co-glycolic acid) (PLGA) scaffold, vascularized tissue formation [17, 18] or adipose tissue [19] was induced by implantation of an arteriovenous loop. Organ patterning, such as fabrication of a parenchymal chamber modeled upon hepatic organ architecture for use as a liver-assist device, has also been reported [20]. In this study, computational models were used to study flow behaviour in human microvasculature, subsequent to which a conceptual device was fabricated. Most recently, the use of three-dimensional computed tomography (CT) scanning and CAD techniques has been used to prefabricate an osseocutaneous flap for autogenous mandible reconstruction using the latissimus dorsi muscle as a vascularized carrier for free tissue transfer [21].

Extending the use of an arteriovenous bundle further via the fabrication of customized vascular channels through microstructural scaffold patterning, together with the use of computational analysis and CAD fabrication of vessel channels, as was done in this study, allows highly accurate fabrication of a biomimetic scaffold potentially allowing prefabrication of large composite osseous-polymer or osteocutaneous-polymer flaps. These could be transferred through microsurgical technique as free flaps with application in reconstructive surgery. 

**Figure 7:**
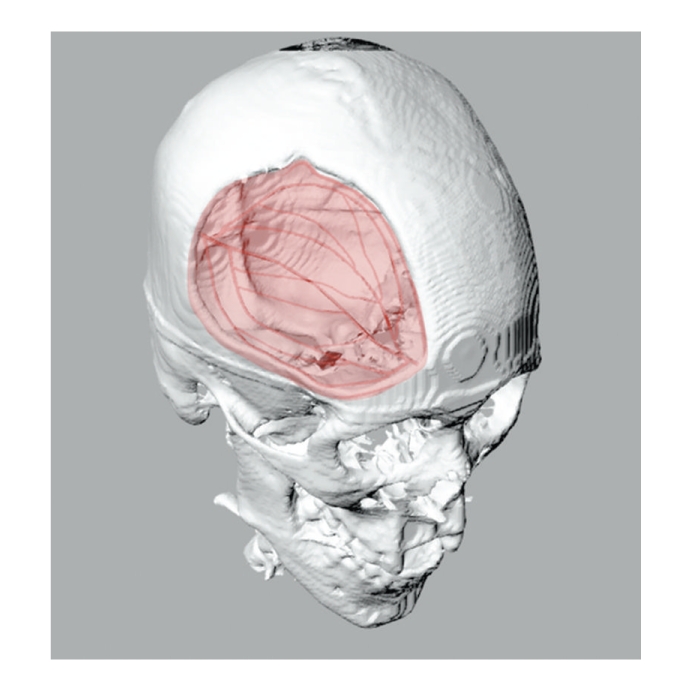
Customized scaffold design with a patient-specific vascular network using computer-aided design (CAD) techniques based on three-dimensional CT data allows modeling and fabrication of a tailored construct. CAD model shown here is tailored to the dimensions and shape of the calvarial defect and also is able to simulate the vessel network in the defect.

While the results in this study are still preliminary, we show that inductive neovascularization through scaffold fabrication techniques is achievable. Hence, this technique, which we would term “*vascular guidance,*” whereby neovascularization is guided through customized channels in a scaffold, might potentially allow fabrication of much larger tissue-engineered constructs than current technologies allow. Our technology also allows fabrication of customized scaffolds with a patient-specific vessel network, allowing tailored construct fabrication based on CT scan data (Figure 7).

In previous studies, we defined the concept of “*cell guidance*” [22, 23], whereby a biomimetic environment is created through constant delivery of cytokines to different areas of an implanted scaffold, so as to allow site-specific homing of cells for purposes of total *in vivo* tissue engineering. Both “*vascular guidance*” and “*cell guidance*” would fall under the concept of “*biological guidance,” *which we define here as “a therapeutic approach for channeling specific biologic events by directing and orchestrating cellular mediators and factors in order to achieve in vivo tissue regeneration.” “*Biological guidance*” represents a shift from current thinking in tissue engineering and organ regeneration but would potentially represent a major step towards clinical translation of tissue engineering techniques.

In conclusion, we show in this proof-of-principle experiment that inductive neovascularization through microstructural scaffold patterning provides a new technique for potentially engineering large cell-scaffold constructs for tissue and organ regeneration, in addition to providing the capability for facilitating reconstructive surgery through free tissue transfer.

## Abbreviations

PLGA: Poly-L-glycolic acid
PCL: Poly-L-caprolactone
CAD: Computer-aided design
HA: Hydroxyapatite
FDA: Fused deposition modeling
PBS: Phosphate-buffered saline
MSC: Mesenchymal stem cells
CT: Computed tomography.
